# Heterosubtypic cross-protection correlates with cross-reactive interferon-gamma-secreting lymphocytes in the ferret model of influenza

**DOI:** 10.1038/s41598-019-38885-0

**Published:** 2019-02-22

**Authors:** Karen E. Gooch, Anthony C. Marriott, Kathryn A. Ryan, Paul Yeates, Gillian S. Slack, Phillip J. Brown, Ross Fothergill, Catherine J. Whittaker, Miles W. Carroll

**Affiliations:** National Infection Service, Public Health England, Porton Down, SP4 0JG United Kingdom

## Abstract

An effective universal vaccine for influenza will likely need to induce virus-specific T-cells, which are the major mediator of heterosubtypic cross-protection between different subtypes of influenza A virus. In this study we characterise the cell-mediated immune response in ferrets during heterosubtypic protection induced by low-dose H1N1 virus infection against an H3N2 virus challenge, given 4 weeks later. Although the ferrets were not protected against the infection by H3N2 virus, the duration of virus shedding was shortened, and clinical disease was markedly reduced. No cross-reactive neutralizing antibodies were detected, but cross-reactive interferon-gamma-secreting T cells were detected in the circulation prior to H3N2 challenge. These T-cells peaked at 11 days post-H1N1 infection, and were strongly induced in blood and in lung following H3N2 infection. The rapid induction of interferon-gamma-secreting cells in ferrets previously infected with H1N1 virus, but not in naïve ferrets, suggests induction of memory T-cells. These results are in accord with the observations that pre-existing cross-reactive T-cells correlate with protection in humans and have implications for outbreak modelling and universal vaccine design.

## Introduction

Influenza viruses are responsible for an estimated 3 to 5 million cases of severe illness and up to 650,000 deaths annually^1^. The widely used inactivated vaccine requires updating every 6 months due to the continual evolution of both influenza A and B viruses by antigenic drift^2^. Influenza A viruses also pose the risk of causing pandemics, in which a novel subtype emerges to which the human population has little or no immunity. Infection with influenza A virus leads to high titres of strain-specific serum antibodies, along with mucosal antibody, and protection from a subsequent infection is mediated by the antibody responses. Infection with influenza A virus also leads to CD4+ and CD8+ T-cell responses, which are essential for clearance of the influenza infection^3–5^. While broadly-neutralizing antibodies can be induced by infection, the great majority of the neutralizing antibody response is directed to strain-specific or subtype-specific epitopes on the viral envelope proteins haemagglutinin (HA) and neuraminidase (NA)^4^. Conversely the T-cell responses are mainly directed against conserved internal antigens such as nucleoprotein (NP) and matrix (M1) proteins^4,5^.

The inactivated vaccine provides strain-specific immunity mediated primarily by neutralizing antibodies, and would not elicit protection in the case of a new pandemic virus. In addition, vaccine failures occur when one or more components of the vaccine are mismatched to the circulating virus strains, either due to failure to predict circulating strains, or due to antigenic changes in the vaccine strains^2,6^. Much research is ongoing into development of “universal” influenza vaccines which would cross-protect between subtypes of influenza A virus. It has long been known that prior infection with one subtype can lead to at least some protection against a different subtype in the ferret model, which is considered the “gold standard” for pre-clinical studies with human influenza A virus^7–9^. The cross-protective immunity appears to involve T-cells^10–12^. These observations are of direct relevance to the human population, as the presence of cross-reactive T-cells has been shown to correlate with protection against disease, both during the 2009 H1N1 pandemic, and in human challenge studies^13–15^. Furthermore, vaccines designed to elicit cross-protective T-cells (targeted to the viral NP, M1 and M2 proteins) are in clinical trials^16–18^.

Studies of cellular immune responses in ferrets have been hampered by the lack of ferret-specific reagents and protocols, compared to the mouse model. Recently such reagents have started to become available, and we and others have demonstrated that the kinetics of the adaptive immune response in ferrets can be studied using techniques such as interferon-gamma (IFN-γ) ELISA and flow cytometry using small blood samples which do not require the ferrets to be culled^19,20^. In this study, we investigated the immune responses involved in cross-protection between H1N1 and H3N2 viruses in the ferret model, using our low-dose challenge model which more closely mimics natural influenza infections than the high infectious doses often used to challenge ferrets^21^. The aim of the study performed here was to investigate the cellular immune response in ferrets following challenge with homologous and heterologous virus strains, to evaluate the role of cellular responses in protection. The H1N1 and H3N2 viruses were chosen as clinically relevant human isolates of the two globally circulating influenza A viruses. In addition, the H1 and H3 proteins are only distantly related phylogenetically, being representatives of HA groups 1 and 2, respectively^22^, thus minimising the possibility of cross-protective antibody responses.

## Results

### Infection with low-dose H1N1 virus induces heterologous protection against subsequent H3N2 virus challenge

**Figure 1:**
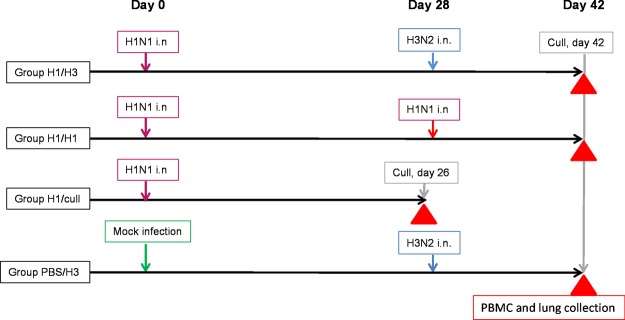
Study design. Each group comprised 6 ferrets. Groups H1/H3, H1/H1 and H1/cull received identical doses of 100 pfu H1N1 virus on day 0. Groups H1/H3 and PBS/H3 received identical doses of 100 pfu H3N2 virus on day 28, whereas group H1/H1 received a second 100 pfu dose of H1N1 virus (homologous challenge). Nasal washes, heparinised blood samples and sera were collected from all animals at time-points specified in Methods. Red triangles indicate collection of PBMC and lung lymphocyte samples. i.n., intra-nasal inoculation.

**Figure 2:**
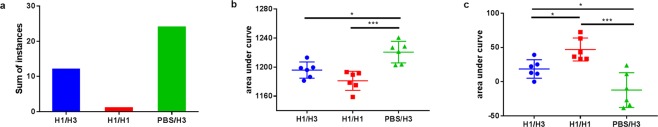
Signs of clinical disease following second virus challenge. Groups H1/H3 and PBS/H3 were challenged with H3N2 virus, group H1/H1 with H1N1 virus. (**a**) frequency of sneezing following challenge, sum for each group; (**b**) AUC for temperature between 50 and 82 hours post-challenge; (**c**) AUC for weight between days 0 and 14 post-challenge. Weights were normalised to weight on day of challenge. In panels b and c, points represent individual ferrets and bars represent group means and standard deviations. Groups were compared by 1-way ANOVA with Holm-Sidak’s multiple comparisons test. *p < 0.05; ***p < 0.001.

The study design is summarised in Fig. 1. Seronegative ferrets were infected intra-nasally (i.n.) with 100 pfu H1N1 virus and allowed to recover from infection. 28 days later 6 ferrets were challenged with 100 pfu H3N2 virus (group H1/H3). As controls, an identical H1N1-infected group (6 ferrets) was challenged with the same H1N1 virus (homologous challenge; group H1/H1), and a mock-infected group (6 ferrets) was challenged with the H3N2 virus (group PBS/H3). An additional group of 6 ferrets was infected with H1N1 virus and subsequently culled at 26 days post-infection to allow assessment of immune responses prior to the day 28 challenge (group H1/cull). Disease post-challenge was assessed on the basis of animal weight, temperature, and clinical scores, and is summarised in Fig. 2. Respiratory signs observed following the second challenge were mainly sneezing, and group H1/H3 showed an intermediate frequency between the control groups H1/H1 and PBS/H3 (Fig. 2a). Fever and weight loss were assessed by calculating areas-under-curves (AUC), and in each case the heterologous group H1/H3 was significantly different from either control group (Fig. 2b,c, Supplementary Figs S4 and S5). Notably, group H1/H3 showed significantly reduced fever (*t*-test, *p* = 0.0043) and weight loss (*p* = 0.013) compared to control group PBS/H3. The homologous challenge group H1/H1 showed weight gain, and no detectable fever.

**Figure 3:**
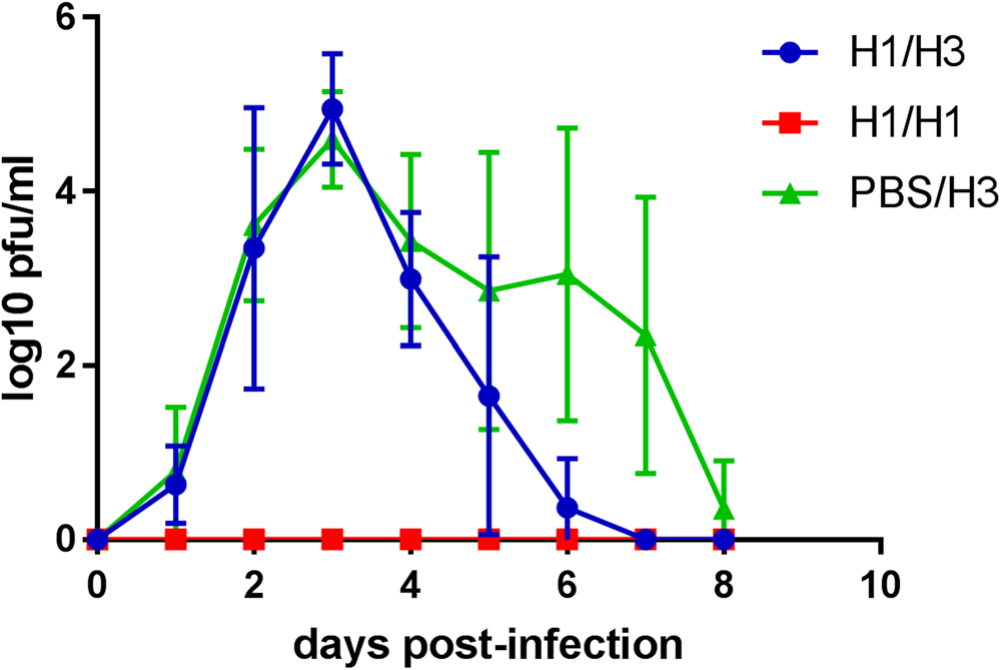
Virus titres in nasal wash following second virus challenge. Points show log mean with standard deviation for each group. Time 0 samples were collected 2 days prior to challenge.

Virus shedding, monitored by plaque assay of nasal wash fluids, was assessed following the H3N2 challenge and results are shown in Fig. 3. Both groups H1/H3 and PBS/H3 showed a peak of shedding 3 days post-challenge, and peak titres were not significantly different. However the duration of shedding was significantly shorter in the heterologous challenge group H1/H3 relative to group PBS/H3 with no prior challenge (Mann-Whitney test, *p* = 0.049). The homologous challenge group H1/H1 showed no detectable virus shedding at any time point (Fig. 3).

Taken together, these data demonstrate that prior infection with a low dose of H1N1 virus was able to fully protect against subsequent challenge with the same virus (no measurable disease or virus shedding), and partially protected against disease caused by a heterologous H3N2 challenge. The duration of H3N2 virus shedding was reduced but not the peak virus titre.

### Inflammatory immune response to heterologous infection

**Figure 4:**
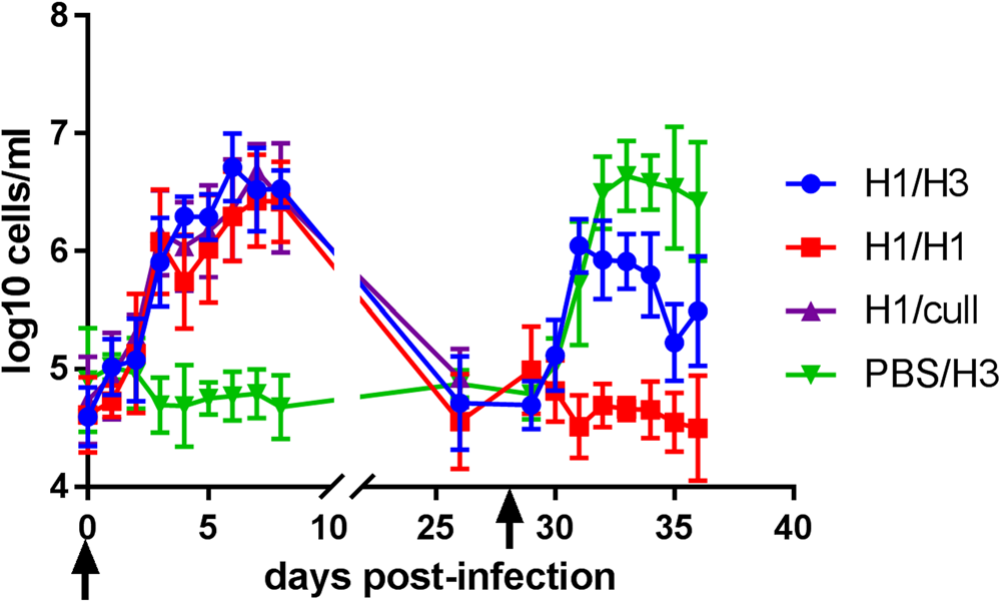
Nasal wash live cell counts. Lines show mean and standard deviation for each group. Ferrets were infected on days 0 and 28 (arrows).

The inflammatory cellular response in the nasal cavity was monitored by counting viable cells in nasal washes. The kinetics of nasal wash cell count over time is shown in Fig. 4. All ferrets infected with H1N1 virus at day 0 showed an increase in cell count of about 2 logs during the first few days after infection, whereas the mock-infected control group PBS/H3 showed no such rise above pre-infection levels. Also as expected, all ferrets infected with H1N1 virus at day 0 showed shedding of infectious virus in nasal washes from days 1–6 (Supplementary Fig. S1) and mock-infected ferrets did not. By 26 days post-infection, nasal wash cell counts had returned to baseline levels (Fig. 4). Upon challenge with H3N2 virus, group H1/H3 showed a rise in nasal wash cell counts, but of significantly lower magnitude than the rise seen in control group PBS/H3 (comparing areas under the curves, 1-way ANOVA, *p* = 0.002). The homologous challenge group H1/H1 showed no rise above baseline (Fig. 4).

### Humoral immune response to primary challenge

**Figure 5:**
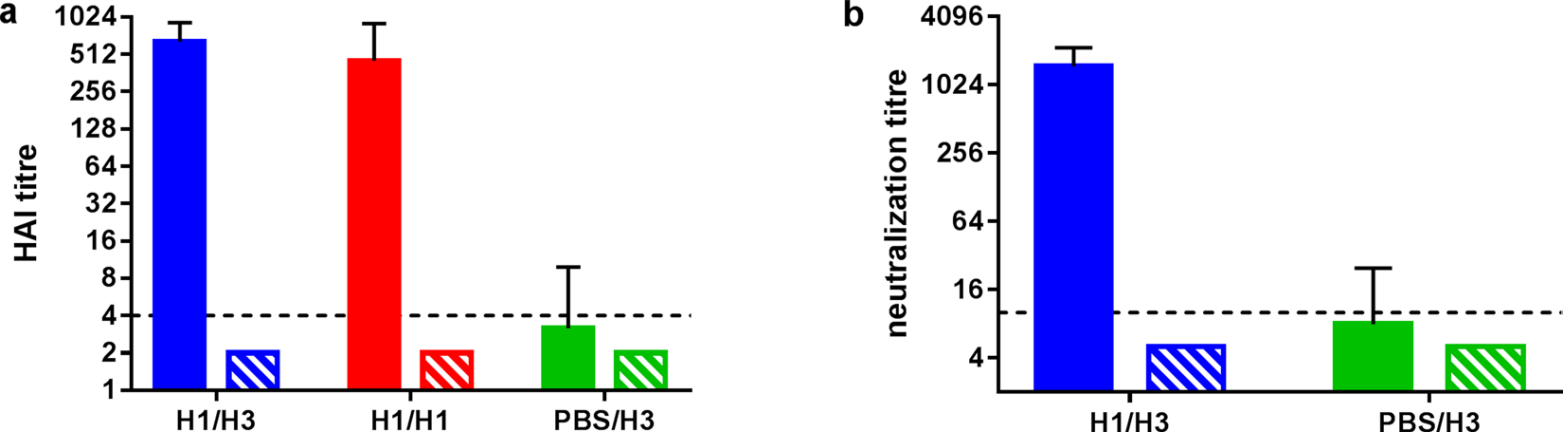
Antibody response to initial infection with H1N1 virus (or PBS control). (**a**) geometric mean HAI titres, (**b**) geometric mean neutralization titres. Limit of detection in a is 4, values < 4 were scored as 2. Limit of detection in b is 10, values < 10 were scored as 5. Error bars show standard deviation. Solid bars show response to H1N1 virus, hatched bars show response to H3N2 virus. Sera were collected 24 days post-infection.

Sera collected 4 days prior to the second challenge were assayed by HAI and microneutralization tests. As expected, groups challenged with H1N1 virus at day 0 all sero-converted, with a geometric mean titre (GMT) of 575 to H1N1 virus on day 24 (Fig. 5a). No HAI titre was detected against the H3N2 virus at this time-point. Mock-infected group PBS/H3 showed no HAI titre against either virus at this time-point. Similarly, group H1/H3 showed a high neutralization titre (≥1280) against H1N1 virus, but no detectable neutralization titre against H3N2 virus (Fig. 5b). Following the second challenge, all H3N2-challenged ferrets sero-converted to H3N2 (GMT 724 for group H1/H3, 1351 for group PBS/H3), whereas the homologous challenge group H1/H1 did not show an increase in H1N1-specific HAI titre (Supplementary Fig. S2).

### Cellular immune response to heterologous and homologous infection

The adaptive cellular immune response was monitored in two ways. The frequency of influenza-specific IFN-ɣ secretion by PBMC and lung mononuclear cells (MNC) isolated at necropsy was assessed using an Enzyme-Linked Immunospot (ELISpot) assay. We also evaluated the quantity of influenza-specific secretion of IFN-ɣ in supernatants created from whole blood collected at regular intervals using an enzyme-linked immunosorbent (ELISA) assay to create longitudinal time courses for each ferret.

**Figure 6:**
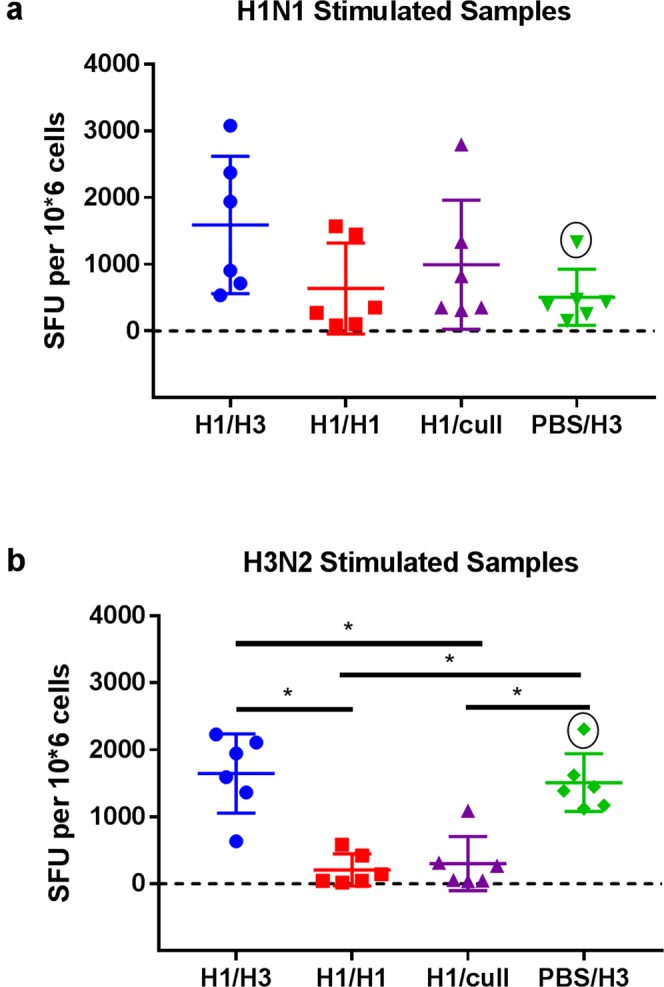
ELISpot titres in PBMC. (**a**) stimulation with H1N1 virus, (**b**) stimulation with H3N2 virus. Points show individual ferrets, with mean and standard deviation overlaid. Groups were compared by Kruskall-Wallis test, using Dunn’s multiple comparisons test. *p < 0.05. The values marked with a circle in group PBS/H3 are from the same ferret.

The frequency of influenza-specific IFN-ɣ producing PBMCs following stimulation with either H1N1 or H3N2 virus is shown in Fig. 6. Responses to H1N1 stimulation were not significantly different between groups H1/H3, H1/H1 and H1/cull (Fig. 6a) (Kruskall-Wallis test, *p* = 0.083). As group H1/cull corresponds to the time-point prior to second challenge, this suggests there was no discernible increase in H1N1-specific IFN-ɣ producing PBMCs following the second H1N1 challenge (group H1/H1) (means 995 and 638 SFU/10^6^ cells, respectively). The presence of IFN-ɣ producing PBMCs able to be stimulated by H1N1 virus in group PBS/H3 (challenged only with H3N2 virus), and of IFN-ɣ producing PBMCs which could be stimulated by H3N2 virus in groups H1/H1 and H1/cull (challenged only with H1N1 virus), suggests the presence of cross-reactive T-cells which are being stimulated by conserved epitopes. Group H1/H3 showed a significant increase in the frequency of H3N2-specific IFN-ɣ producing PBMCs compared to group H1/cull (from mean 302 to 1648 SFU/10^6^ cells, *p* = 0.026), to a similar level as seen in H3N2 control group PBS/H3 (mean 1512 SFU/10^6^ cells) (Fig. 6b).

**Figure 7:**
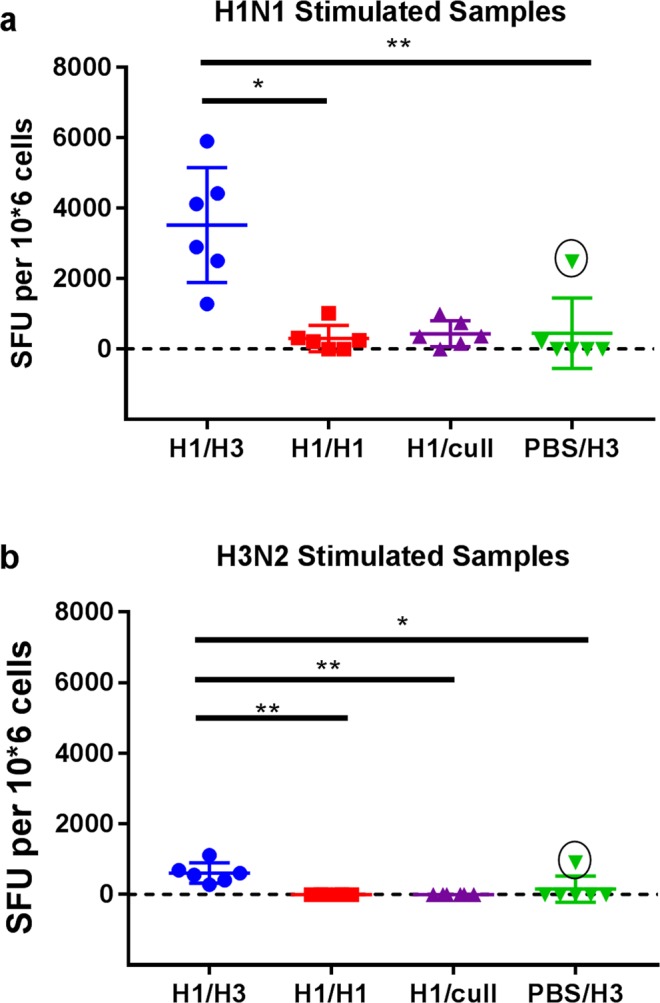
ELISpot titres in lung MNC. (**a**) stimulation with H1N1 virus, (**b**) stimulation with H3N2 virus. Points show individual ferrets, with mean and standard deviation overlaid. Groups were compared by Kruskall-Wallis test, using Dunn’s multiple comparisons test. *p < 0.05, **p < 0.01. The values marked with a circle in group PBS/H3 are from the same ferret as highlighted in Fig. 6.

The frequency of lung MNCs producing IFN-ɣ in response to virus stimulation differed to those observed in PBMCs (Fig. 7). Groups H1/H1, H1/cull and PBS/H3 showed a low frequency of influenza-specific IFN-ɣ producing lung MNC (mean ≤ 450 SFU/10^6^ cells) when stimulated with H1N1, and no response to H3N2-stimulation (except 1 ferret in group PBS/H3). However, heterologous challenge group H1/H3 showed significant increases in both H1N1 and H3N2-specific IFN-ɣ producing lung MNC compared to the other groups (Fig. 7; Kruskall-Wallis tests, *p* < 0.01). It was noted that the outlier in group PBS/H3 in Figs 6 and 7 (circled) was the same individual ferret.

**Figure 8:**
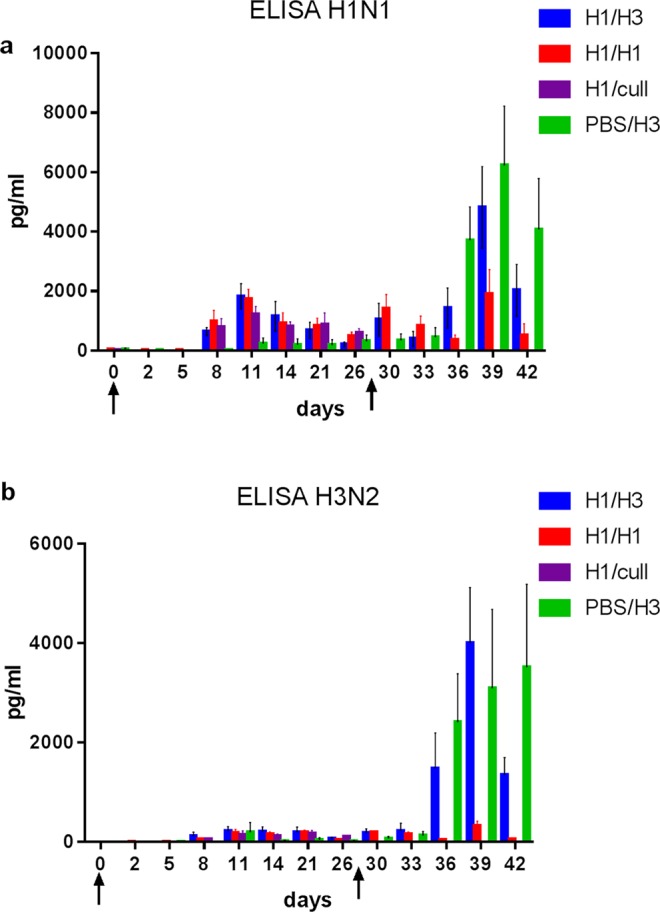
Whole blood IFN-γ ELISA. Bars show group mean values in pg/ml following stimulation with (**a**) H1N1 virus and (**b**) H3N2 virus. Arrows indicate the timing of the first and second infections, at days 0 and 28. Error bars show standard error of the mean. Upper limit of detection was 12000 pg/ml.

The quantity of influenza-specific IFN-ɣ production was also determined by ELISA in supernatants created from virus stimulated heparin blood samples collected at regular intervals throughout the study. We have previously shown that low-dose infections with H1N1 and H3N2 viruses in the ferret model produce similar influenza-specific IFN-γ production profiles in blood^19^. In this study, responses after the initial H1N1 challenge were comparable in each group H1/H3, H1/H1 and H1/cull with IFN-ɣ detectable by 8 days post-infection and the peak H1N1-specific IFN-ɣ production at 11 days post-infection, followed by a fall in response up to day 26 post-infection (Fig. 8a). In particular, the day 11 peak for groups H1/H3, H1/H1 and H1/cull had a mean value of 1210–1827 pg/ml, compared to group PBS/H3 and pre-challenge samples which were <230 pg/ml. By day 26 the mean values for groups H1/H3, H1/H1 and H1/cull had fallen to 213–590 pg/ml. The influenza-specific IFN-γ production profile following the first challenge (up to day 26), measured by area under the curve for each ferret, was not significantly different between groups H1/H3, H1/H1 and H1/cull, but each produced significantly more influenza-specific IFN-γ than mock-infected group PBS/H3 (Supplementary Fig. 3; Mann-Whitney test, *p* < 0.04). Lower levels of cross-reactive H3N2 –specific IFN-ɣ could also be detected with similar kinetics following the H1N1 challenge (Fig. 8b), of approximately 10-fold lower level than the H1N1-specific response. Mock-infected group PBS/H3 did not show a measurable response prior to the day 28 H3N2 challenge (with the exception of one of the six ferrets; Supplementary Fig. 3). Following heterologous challenge on day 28, a peak of influenza-specific IFN-γ production was observed in group H1/H3, responding to stimulation by both H1N1 and H3N2 viruses (mean 4811 and 4002 pg/ml, respectively), at 11 days post-infection (Fig. 8a,b). The H1N1-specific IFN-ɣ secretion was detectable in most animals by 2 days post-infection. A similar response after H1N1 stimulation was observed in homologous challenge group H1/H1, although with a lower peak at day 11 (mean 1914 pg/ml; Mann-Whitney test, *p* = 0.045). The H3N2 control group PBS/H3 showed responses to both viruses peaking at 11–14 days post-challenge. The H3N2-specific IFN-ɣ secretion was of a similar magnitude in groups H1/H3 and PBS/H3, and both were significantly greater than the H3N2-specific IFN-ɣ response observed in group H1/H1 (Mann-Whitney test, *p* = 0.002), as determined by comparing areas under the curves (Supplementary Fig. 3). The early (2 days post-challenge) response to H1N1 virus was not observed with H3N2 stimulation (Fig. 8b).

## Discussion

The data presented here demonstrate that infection with a low dose of H1N1 virus 28 days prior to infection with H3N2 virus leads to significant partial protection against disease (weight loss, fever and sneezing), and reduced duration of virus shedding. This same low-dose infection with H1N1 led to complete protection against disease and virus shedding following a homologous H1N1 challenge. This complete protection was accompanied by high serum HAI and neutralization titres against H1N1 virus prior to the second challenge. These antibody responses were a clear correlate of, and presumably mediated, the protection. However, no detectable HAI or neutralization titres were observed against H3N2 virus, so the partial protection seen in the heterologous challenge group was most likely not mediated by antibody responses. The ferrets culled 26 days after the initial H1N1 challenge (group H1/cull), i.e. prior to the second challenge, showed influenza-specific IFN-γ producing PBMCs after stimulation with H1N1 and H3N2 virus (Fig. 6). These cells could be cytotoxic CD8+ cells, Th1 CD4+ cells, or both; we did not define their phenotype in this study. Regardless, the presence of influenza specific PBMCs able to produce IFN-ɣ when stimulated with H3N2 prior to the heterologous H3N2 challenge suggests that the partial protection observed was mediated at least in part by these cells. By analogy with T-cells in influenza-infected humans and mice, it is likely that a proportion of the T-cells were able to recognise conserved epitopes, for example in the highly conserved NP and M1 proteins^14,23^. Following the H3N2 challenge, a significant increase in H3N2-specific IFN-γ producing PBMCs was observed; this was accompanied by notable increases in H1N1-specific and H3N2-specific IFN-ɣ producing lung MNCs (Fig. 7). These responses in the lung were significantly higher than those measured after the primary H3N2 infection (group PBS/H3), suggesting that they were influenza-specific memory T-cells able to recognise conserved antigens in the H1N1 virus. ELISpot responses in both lung lymphocytes and PBMCs were negatively correlated with the duration of virus shedding, further suggesting a role for these cells in clearance of virus infection (Spearman rank correlation, R = −0.67 to −0.89, p = 0.0004 to 0.02). For the heterologous challenge group H1/H3, it was noted that the mean ELISpot signal was ~5-fold higher when stimulated with H1N1 virus, compared to stimulation with H3N2 virus (Fig. 7). We do not believe that this is due to a lack of sensitivity in the H3N2-stimulated assay, as PBMC ELISpot counts can be as high in H3N2-infected ferrets as in H1N1-infected ferrets (Fig. 6 and ref.^19^). In addition, this effect is not seen in PBMC samples (Fig. 6). A more detailed investigation of the antigen-specificity of the lung lymphocytes may shed new light on this observation.

Influenza-specific IFN-ɣ was measured in stimulated blood by ELISA, responses were observed by 8 days after the first H1N1 infection (Fig. 8). The low-dose H3N2 infection showed similar kinetics (Fig. 8b), in agreement with earlier studies^19^. In contrast, the second challenge in groups H1/H3 (heterologous challenge) and H1/H1 (homologous challenge) led to an increase in influenza-specific IFN-γ production by 2 days post-challenge, in at least some of the ferrets. This rapid response was not observed when naïve ferrets were challenged with H3N2 virus (group PBS/H3), suggesting a rapid effector memory response. The peak response following the second challenge was significantly lower in the homologous challenge group than in the heterologous challenge group to H1N1 stimulation (Mann-Whitney test, *p* = 0.045), and was significantly lower to H3N2 stimulation (*p* = 0.0051). Taken together with the ELISpot data, this suggests that H3N2 challenge (but not homologous H1N1 challenge) induced rapid proliferation of a subset of memory T-cells which recognised the H3N2 antigens.

Following the homologous challenge (group H1/H1), the only immune response that was detected was H1N1-stimulated IFN-γ production in whole blood: there was no increase in HAI titre, nasal wash cell counts, or the frequency of influenza-specific IFN-ɣ producing PBMC or lung MNC following the second challenge. The amount of virus shed from the nasal cavity was also undetectable, indicating that the immune memory (presumably antibodies present in the nasal cavity) was able to suppress virus replication sufficiently to limit, but not entirely eliminate, the immune response to the homologous challenge. By contrast, the heterologous H3N2 challenge led to unsuppressed virus replication for at least 3 days post-infection, and induction of a memory immune response (presumably T-cells) which was then able to control virus replication, leading to a reduction in virus shedding and disease.

In order for the ferret to remain a gold standard model for human influenza infection, it is important that protective immune responses other than neutralizing antibodies can be demonstrated. This study has confirmed that heterosubtypic cross-protection can be reliably modelled in ferrets and that cellular immune responses can be quantified. The results presented here are consistent with the reports that pre-existing cross-reactive memory T-cells protect against disease in human studies. The cell type which correlated with protection was reported to be CD4+ T-cells in a volunteer challenge study^14^, and CD8+ T-cells in a cohort study following natural infections in the 2009 H1N1 pandemic^13^, in both cases with T-cells directed against the conserved NP and M1 proteins being important. It would be interesting to further characterise the T-cell responses in the ferret model to determine how well it can reproduce what is observed in humans, for example by identifying the major responsive epitopes using panels of peptides. A recent study has shown that ferrets challenged with high doses of H1N1pdm or H3N2 viruses produce cross-reactive CD4+ and CD8+ T-cells, mainly targeted to the internal proteins of the virus^24^. As the existing inactivated vaccine is a very poor inducer of T-cell immunity in both humans and ferrets^5,25^, the induction of cross-reactive T-cell responses is an important property for so-called universal influenza vaccines under development^26^. As the currently licensed live attenuated vaccines can induce T-cells^27,28^, it will be important to determine the potential for heterosubtypic cross-protection induced by these vaccines in the ferret model.

## Methods

### Viruses and cells

Influenza A/California/04/09 (H1N1) and A/Perth/16/09 (H3N2) were obtained from the Centers for Disease Control and Prevention (CDC, Atlanta, USA), and National Institute for Biological Standards and Control (NIBSC, Potters Bar, UK), respectively. Viruses were propagated and titrated in Madin-Darby Canine Kidney (MDCK) cells, obtained from European Collection of Cell Cultures (ECACC, Porton Down, UK). Virus titres were determined by plaque assay on MDCK cells under an agar overlay, followed by staining with crystal violet.

### Animals

24 female ferrets (*Mustela putorius furo*) were obtained from Highgate Farm, UK, and confirmed as seronegative for influenza H1N1pdm09, H3N2 and influenza B antibodies by haemagglutination-inhibition assay before experiments commenced. Mean weight at initial challenge was approximately 930 g. An identifier chip (Bio-Thermo Identichip, Animalcare Ltd, UK) was inserted subcutaneously into the dorsal cervical region of each animal. Animals were monitored for signs of disease twice daily (approximately 8 hr apart), and weight was recorded daily. Temperature was monitored twice daily using the chip, increased to 7 times daily during the 3 days following virus challenges, to ensure any peak of fever was recorded. Animals were sedated by intramuscular injection of ketamine/xylazine (17.9 mg/kg and 3.6 mg/kg bodyweight), prior to intranasal instillation of challenge virus (0.2 ml total, 0.1 ml per nostril) diluted in phosphate buffered saline (PBS). Nasal washes were obtained using 2 ml PBS. The experimental animal work described here was scrutinized and approved by the Animal Welfare and Ethical Review Body of Public Health England (Porton), as required by the UK Home Office Animals (Scientific Procedures) Act, 1986. The premises in which the work was conducted are approved under Home Office Certificate of Designation PCD70/1707. All methods involving ferrets were performed in accordance with the relevant guidelines and regulations.

### Serum antibody

Serum samples were titrated by haemagglutination-inhibition assay (HAI) using 4 HA units per well of the relevant virus, followed by addition of chicken red blood cells. Selected sera were also titrated by microneutralization assay on MDCK cells.

### Isolation of Peripheral Blood Mononuclear Cells

Buffy coats containing lymphocytes were prepared from fresh heparin anti-coagulated blood by density separation on Histopaque 1083^19^. Cells were collected by centrifugation and re-suspended in an appropriate volume of cryomedia allowing the cells to be frozen in liquid nitrogen in 1 ml aliquots at a concentration of 3 × 10^6^ to 1.3 × 10^7^ cells/ml.

### Isolation of Lung Mononuclear Cells

Dissected lungs were dissociated as described previously^19^. The tissue solution was passed through two cell sieves (100 µm then 70 µm) and mononuclear cells were purified and frozen as described for PBMCs above.

### Red Blood Cell Removal

Red blood cells were removed from PBMC and lung MNC preparations using ACK Lysing Buffer^19^ (Gibco, ThermoFisher Scientific, UK).

### Viable Cell Counts

Viable cells in nasal washes, PBMCs and lung MNCs were counted using a NucleoCounter®NC-200 (ChemoMetec, Allerod, Denmark).

### Interferon-gamma (IFN-γ) ELISpot Assay

PBMC and lung MNC were assayed as described previously^19^. In brief, PBMC and lung MNC were assessed for responses to A/California/07/2009 (H1N1) and A/Perth/16/2009 (H3N2). Both viruses were used at an MOI of 0.08. Following overnight stimulation, IFN-γ expressing cells were detected using the Mabtech Ferret interferon-gamma ELISpot kit with pre-coated plates (Mabtech, Nacka, Sweden). Results from duplicate tests were averaged. Data were analysed by subtracting the mean number of spots in the control wells (cells and allantoic fluid) from the mean counts of spots in wells with cells and antigen.

### Ferret IFN-γ enzyme linked immunosorbent assay (ELISA)

Heparinised whole blood was diluted 1:10 with serum free RPMI 1640 medium and incubated with either A/California/07/2009 (H1N1) or A/Perth/16/2009 (H3N2) allantoic fluids (1.6 × 10^6^ pfu). Phytohemagglutinin PHA-M (PHA) (Sigma-Aldrich, Dorset, UK) was used as a positive control and egg allantoic fluid was used as a negative control. Blood was stimulated for 4 days at 37 °C, after which plasma supernatants were collected and cryopreserved at −80 °C. The Ferret IFN-γ ELISA Development Kit (ALP) (Mabtech, Nacka, Sweden) was used to determine the quantity of IFN-ɣ secreted by cells in the blood as described^19^.

### Statistical Analysis

Statistical analyses were performed with GraphPad Prism 7 (GraphPad Software, La Jolla, CA), SigmaPlot 12.0 (Systat Software Inc), and Minitab 16 (Minitab Inc). *p*-values of <0.05 were considered significant.

## Supplementary information

Supplementary Information

## Data Availability

All data generated or analysed during this study are included in this published article (and its Supplementary Information files).
