# Supplementary material for: Heterosubtypic cross-protection correlates with cross-reactive interferon-gamma-secreting lymphocytes in the ferret model of influenza
Source: Sci Rep. 2019 Feb 22;9:2617. doi: 10.1038/s41598-019-38885-0 (PMC6384896; doi:10.1038/s41598-019-38885-0)
Supplement: Supplementary file 1 — Supplementary Information [file 41598_2019_38885_MOESM1_ESM.pdf]

Supplementary information:

Heterosubtypic cross-protection correlates with cross-reactive interferon-gamma-secreting lymphocytes in the ferret model of influenza

Karen E. Gooch, Anthony C. Marriott, Kathryn A. Ryan, Paul Yeates, Gillian S. Slack, Phillip J. Brown, Ross Fothergill, Catherine J. Whittaker and Miles W. Carroll.

Supplementary Figures S1-S5

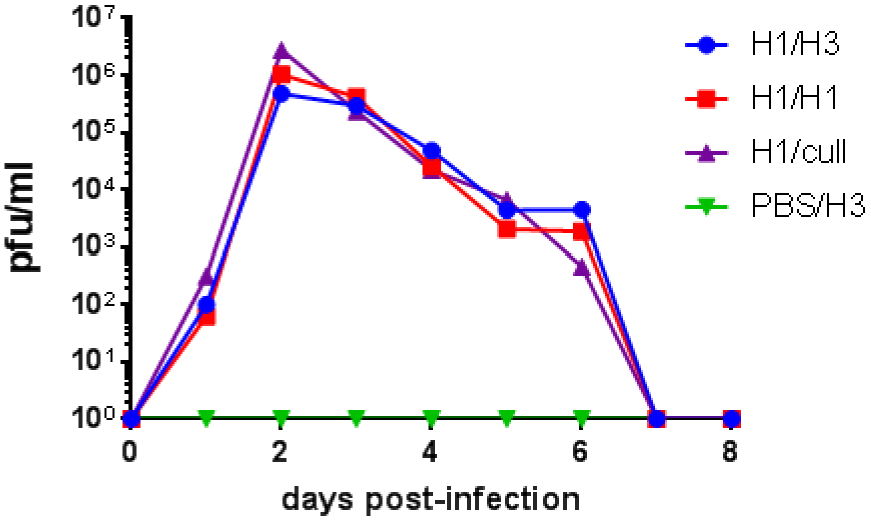

Supplementary Fig. S1. Virus titres in nasal wash following initial challenge. Lines show geometric mean plaque titre for each of the groups.

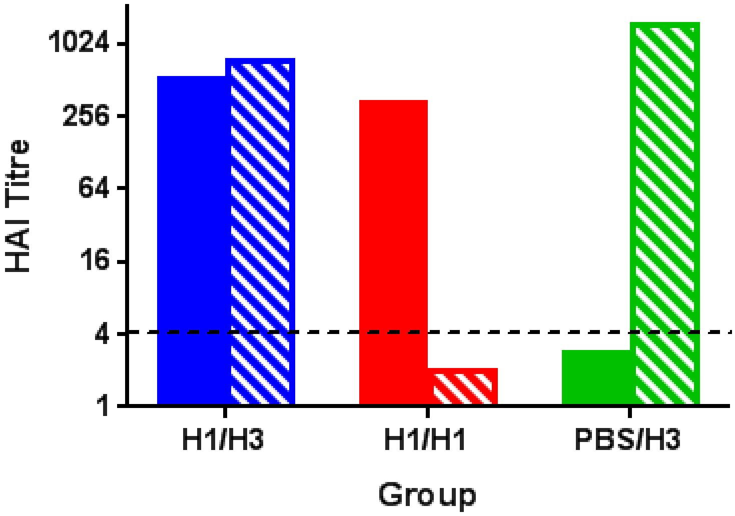

Supplementary Fig. S2. Group mean HAI titres following second challenge. Groups H1/H3 and PBS/H3 were challenged with H3N2 virus, group H1/H1 with H1N1 virus. HAI titres were determined using H1N1 virus (solid bars) and H3N2 virus (hatched bars). Limit of detection is 4 (dotted line), values < 4 were scored as 2.

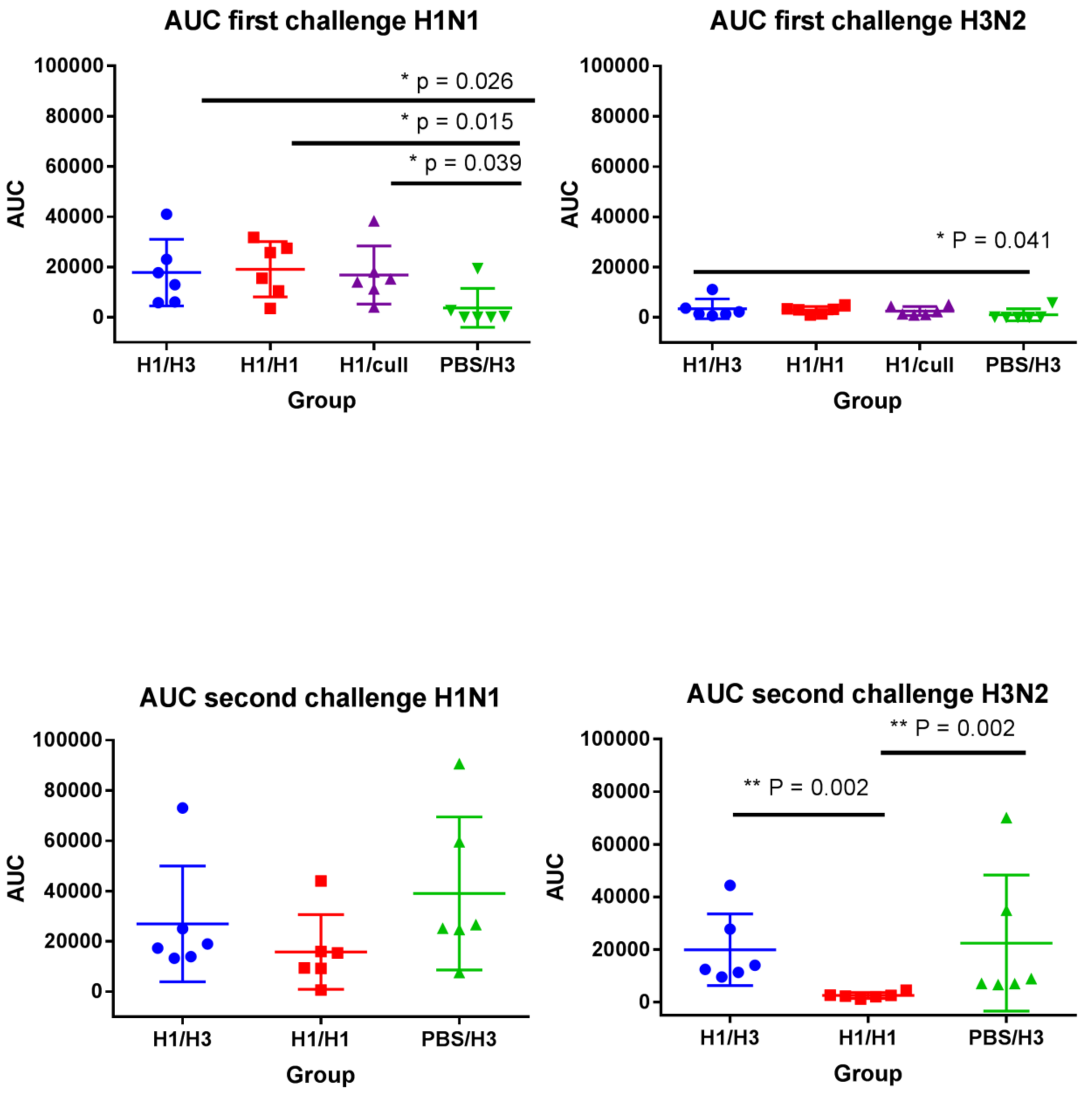

Supplementary Fig. S3. Area under curve (AUC) values for IFN-γ ELISA time-courses for individual ferrets. First challenge comprises days 0 to 26; second challenge comprises days 30 to 42 i.e. following the day 28 challenges. H1N1 and H3N2 refer to the virus used to stimulate the blood samples.

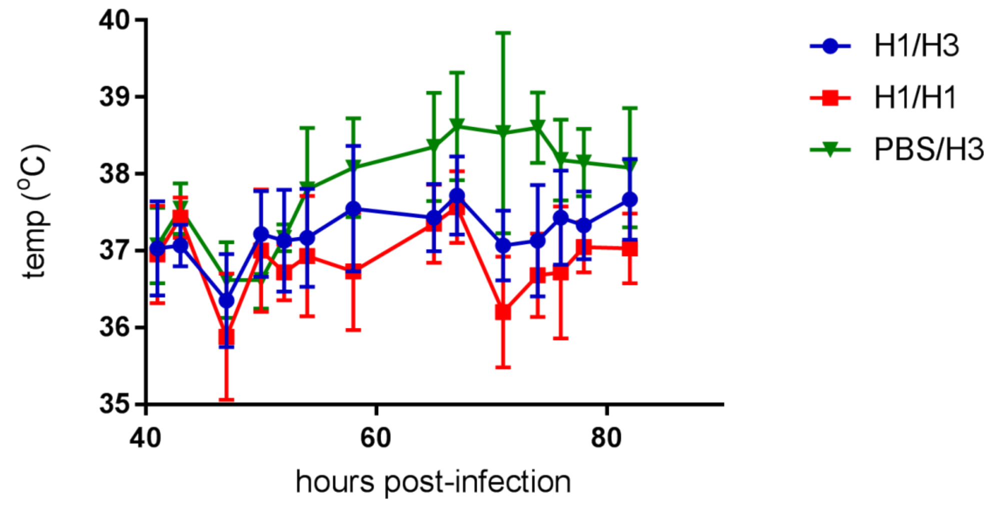

Supplementary Fig. S4. Ferret temperatures following second challenge. Groups H1/H3 and PBS/H3 were challenged with H3N2 virus, group H1/H1 with H1N1 virus. Error bars show standard deviation.

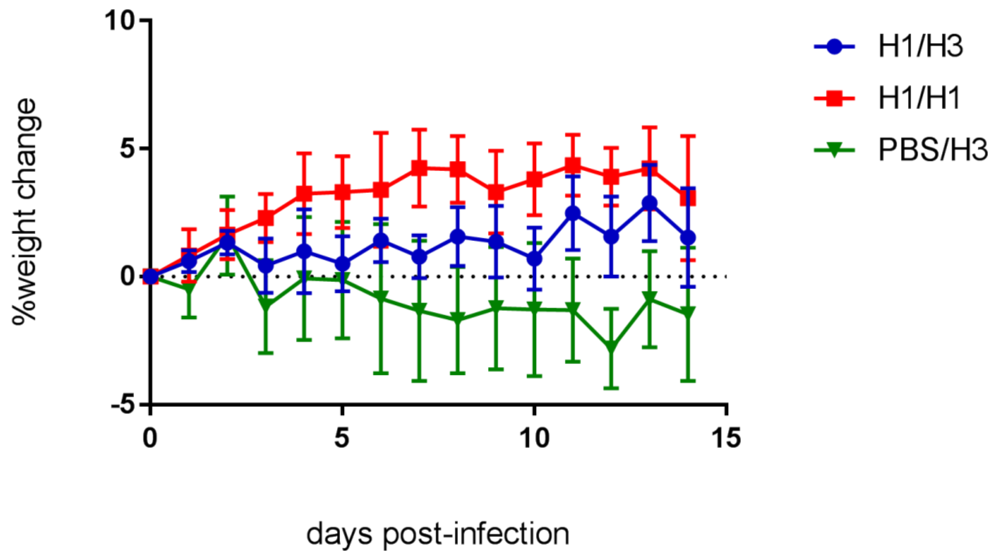

Supplementary Fig. S5. Group mean weight change following second challenge. Groups H1/H3 and PBS/H3 were challenged with H3N2 virus, group H1/H1 with H1N1 virus. Weights were normalised to weight on day of infection. Error bars show standard deviation.
